# Neurons and Glia Cells in Marine Invertebrates: An Update

**DOI:** 10.3389/fnins.2020.00121

**Published:** 2020-02-18

**Authors:** Arturo Ortega, Tatiana N. Olivares-Bañuelos

**Affiliations:** ^1^Laboratorio de Neurotoxicología, Departamento de Toxicología, Centro de Investigación y de Estudios Avanzados del Instituto Politécnico Nacional, Mexico City, Mexico; ^2^Instituto de Investigaciones Oceanológicas, Universidad Autónoma de Baja California, Ensenada, Mexico

**Keywords:** neuronal cells, marine models, neurogenesis, invertebrate neuroregeneration, marine neurophysiology

## Abstract

The nervous system (NS) of invertebrates and vertebrates is composed of two main types of cells: neurons and glia. In both types of organisms, nerve cells have similarities in biochemistry and functionality. The neurons are in charge of the synapse, and the glial cells are in charge of important functions of neuronal and homeostatic modulation. Knowing the mechanisms by which NS cells work is important in the biomedical area for the diagnosis and treatment of neurological disorders. For this reason, cellular and animal models to study the properties and characteristics of the NS are always sought. Marine invertebrates are strategic study models for the biological sciences. The sea slug *Aplysia californica* and the squid *Loligo pealei* are two examples of marine key organisms in the neurosciences field. The principal characteristic of marine invertebrates is that they have a simpler NS that consists of few and larger cells, which are well organized and have accessible structures. As well, the close phylogenetic relationship between Chordata and Echinodermata constitutes an additional advantage to use these organisms as a model for the functionality of neuronal cells and their cellular plasticity. Currently, there is great interest in analyzing the signaling processes between neurons and glial cells, both in vertebrates and in invertebrates. However, only few types of glial cells of invertebrates, mostly insects, have been studied, and it is important to consider marine organisms’ research. For this reason, the objective of the review is to present an update of the most relevant information that exists around the physiology of marine invertebrate neuronal and glial cells.

## Introduction

Marine organisms have been used as study models in different branches of the biological sciences like toxicology, evolutionary biology, developmental biology, biomedicine, and neurobiology. The use of these unique models has allowed identifying diverse mechanisms related to the functioning of the nervous system (NS). Multicellular living organisms have a NS composed of a variety of cellular types. The proteins and macromolecules of these different and specialized cells cooperate between them to get a functional NS which coordinates the major body functions in organisms (Arendt, 2015). Cells of nervous tissue are in control too of organism’s response, and its reaction to environmental stimulus. Understanding brain activities would be relevant in the biomedical field for the identification and treatment of neuropathologies, including those present in humans.

Nerve cells existed and are found in both multi and single-celled marine organisms where, as in the rest of the organisms, arrangement of nerve cells and communication between them is the key to understand how all NSs process, select, and transport information, and even how these cells are regenerated. Among all the organisms, invertebrates have the simpler NS structured with few and large cells, organized in a simple and accessible way (Blankenship and Houck, 2014). In general, invertebrate NS are composed of spherical, ellipsoidal or cylindrical lobes of tissue (tissue shapes frequently are directly related to the observation method), while vertebrate NS tissue is larger, with specific structural and physiological characteristics, that requires specific methods to be observed (Leise, 1990). Invertebrate and vertebrate NS consist of two types of cells named neurons (mainly motor, sensory, and interneurons), and glia cells. Despite differences, the invertebrate nervous cells present the same biochemistry and functionality characteristics as those of vertebrates, demonstrating the high degree of phylogenetic conservation between phyla. Nevertheless, there are differences in cellular properties and cellular arrangements of neural areas among phyla.

Differences between phyla are relevant to understand the origin, development, and regeneration of marine NS. Origins of NS remain unresolved until now (Moroz et al., 2014), but the discovery and identification of key basic proteins in marine unicellular organisms is providing relevant information in this regard (Alié and Manuël, 2010; Hoffmeyer and Burkhardt, 2016; Cavalier-Smith, 2017). Developing mechanisms of NS are better understood where cellular division, differentiation, and cellular migration are relevant processes required in both vertebrates and invertebrates (Anderson et al., 1980; Bulloch, 1985). Respect to the regeneration processes in the NS of marine organisms, the process have been observed for years (Weis and Weis, 1978; Ladurner et al., 2000; Dahlberg et al., 2009), and now some key molecules are used as regeneration factors (Bailly et al., 2014; Joly, 2017; Kamenev and Dolmatov, 2017).

In the following sections, the prevailing information regarding neurons and glial cells of the NS in the main families of marine invertebrates will be described.

## Nervous System in Marine Organisms

The study of NS in marine organisms has been done using organisms with an easily accessible and identifiable system. It has also been considered to those organisms that have a system with conserved characteristics to that of higher mammals, such as humans. In this group of organisms the main representatives are the sea slug *Aplysia californica* and the squid *Loligo pealei*, whose studies have been the basis of relevant research that led to the winning of two Nobel prizes in Physiology and Medicine (in 1963 and in 2000, respectively) (Nobel, 2019).

The NS of marine organisms is made up of specialized and organized cells (with particular shapes and functions) that act together to control the organism behavior, and to sense and respond to environmental stimuli (Northcutt et al., 2017). NSs are expressed as nerveplexus, dorsal nerve cord, and ventral nerve cord. Nerve cells interact between them at specific points and under certain conditions to perform all the organism functions. The NS gives organisms the capability to respond to environmental stimuli by receiving (internal or external), encoding, transmitting, and processing information (Hickman et al., 2008). Ultrastructural examination demonstrates that synapses are present in vertebrates and invertebrates, with common mechanisms like changes in ions concentrations and transmitter release (Watson, 1992).

The invertebrate neuronal cells are in simple numerical proportions, with a diverse composition and organization that includes large neurons (Meinertzhagen, 2017). They are on charge of the electric signaling transmission (or synapses) through between them, and among distinct body parts. On the other hand, glia cells participate in the early development of the NS, as support and protection of neurons, in the maintenance of axon function for normal neurotransmission, in proportionate the normal supply of metabolic fuel to the neurons, and in the homeostatic regulation of the NS (Denes et al., 2007; Coles, 2009; Brand and Livesey, 2011). Besides, glia cells participate too as a blood–brain barrier, and in the guidance mechanisms of young neurons to ensure their appropriate migration to specific targets (Leise, 1990; Coles, 2009).

**FIGURE 1:**
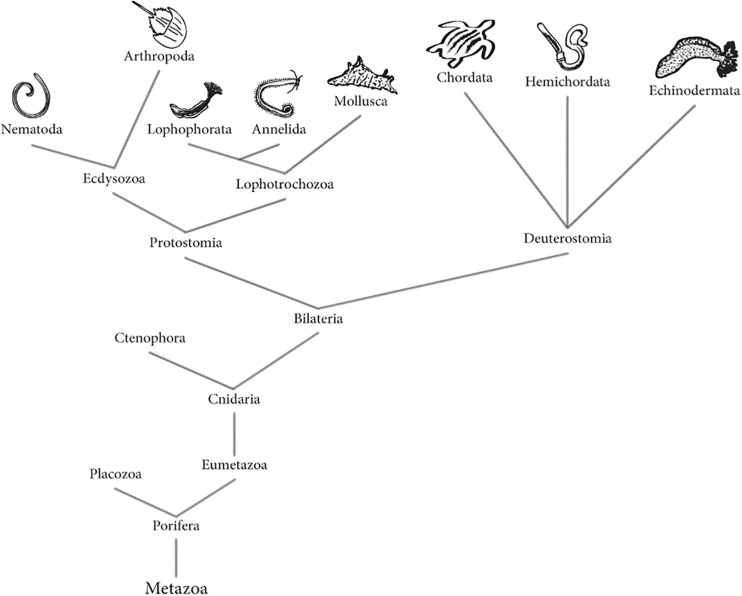
Animal (Metazoa) phylogenetic tree. Tree represents both the morphological and molecular phylogeny between animals, according to recent evidence. Phylogenetic tree based on information from Ghorai and Ghosh (2014), Isaeva et al. (2014), Whelan et al. (2015), Brusca et al. (2016) and Fröbius and Funch (2017).

To elucidate the evolutionary origin of NS in marine organisms, the comparison of the types of constituent neuronal cells is required. Evolution of metazoan phylogeny (Figure 1), from nerve nets to centralized NS has been the subject of discussion. Nerve net concept is referred to the simplest organization of a NS, where signals are transmitted in any direction; recently this term includes irregular arrays of neurites that come from monopolar, bipolar or multipolar neurons (Hejnol and Rentzsch, 2015). NS of metazoan phylogeny has been examined to find the evolutionary relationship between nerve nets and centralized NSs, as well as the possible dorsoventral axis inversion in vertebrates, and the common brain structure of bilaterian and chordate ancestry (Meinertzhagen and Okamura, 2001; Cañestro et al., 2005; Lowe et al., 2006). Important discoveries have been made thanks to the identification of key basic proteins in the unicellular marine organisms, the Choanoflagellates, organisms whose brains and nerve cells have been studied with evolutionary and functional proposes (Alié and Manuël, 2010). Studies in the annelid marine worm *Platynereis dumerilii* have concluded that the protostome and deuterostome ancestor had a centralized NS. This would explain why chordates and annelids share similarities in their NS pattering (Telford and Littlewood, 2009).

In Protostomia clade, the larval apical organ and adjacent lateral cells originate the brain (Lacalli, 1984), where neurons are identifiable individually. It is suggested that in these organisms, neurogenesis is a precise and controlled process (Harzsch and Müller, 2007). Molecular, morphological, and embryological evidence suggest that chordates are inverted protostomes (Arendt and Nübler-Jung, 1994; Nübler-Jung and Arendt, 1996), and its ventral nerve cord is comparable with the dorsal NS of vertebrates (Nielsen, 1999). Thanks to Protostomia marine organisms like *Sagitta setosa* (Harzsch and Müller, 2007), *Spadella cephaloptera* (Perez et al., 2013), and *Symsagittifera roscoffensis* (Semmler et al., 2010) it has been possible to get a better understanding about the evolutionary development of the NS centralization, or in other words, from an irregular and diffuse nerve array to a centralized nerve organization. It is important to mention that the evolutionary transition of nerve networks to the centralization of the NS involved both a localized concentration of neurons and their interrelation to function in a coordinated manner (Arendt et al., 2008).

Neural anatomy of deuterostomes including Echinodermata, present a plexus like NS meanwhile the chordates exhibit a truly dorsal NS with parts of the common ancestor (Byrne and Cisternas, 2002; Cañestro et al., 2005). The marine arrow worm Chatognatha is an example of an organism with ancestral plexus and centralized nervous structures (Harzsch and Wanninger, 2010). It is speculated that these organisms are the earliest protostome, and its study contributes to the understanding of the NS evolution in Bilateria (Fröbius and Funch, 2017; Nielsen, 2017).

In neurobiology, marine Nematoda, Annelida, and Mollusca phylum are among the most studied Protostomia organisms. In the clade Deuterostomia most studied are Chordata and Echinodermata, but Hemicordata is taking importance since the development of their NS can lead to understand the evolution of the system in all organisms. In the following sections, updated information regarding the NS of the mentioned invertebrate phylum will be described. Table 1 summarizes the known main characteristics for each Phylum in terms of development, neurogenesis, anatomy, and physiology.

**TABLE 1:** Comparative analysis of marine invertebrate’s nervous system.

Phylum	Development/Neurogenesis	Adult anatomy	Physiology	References
Nematoda	Neurons are mainly formed by the AB-lineage (95% approximately) Neurogenesis involves various types of intrinsic factors that are sent to neurons through the process of molecular asymmetric divisions	Throughout almost all species is made up of a nerve ring, constituted of four ganglia and nerve cords Set up by few hundreds of neurons (194–302) Is constantly changing according to the organism functional necessities Two glial cells are present in some species with processes of 0.3–0.9 μm thick	Constitute sensory cells, serve as tactile receptors, and monitor bending of the body during locomotion	Teuchert, 1977; Houthoofd et al., 2003; Blaxter, 2011; Hartenstein and Stollewerk, 2015; Brusca et al., 2016
Arthropoda	Is formed from clusters of neuroectodermal cells Most neurons are generated from neuroblasts Neurogenesis in the species suggested that the process is regulated by a common and ancestral mechanism	Constituted by the dorsal brain, and by a ventral, longitudinal and ganglionated nerve cord Involves the distinct sensillia (sense) organs like leg tips, joints, mouthparts, and antennae	Set up of photoreceptors (simple ocelli, complex lensed ocelli, and compound eyes), and sensillia organs Establish communication between new sensillia organs and the old ones, in each molt	Laverack, 1987; Elofsson and Hessler, 2005; Sandeman et al., 2011; Hartenstein and Stollewerk, 2015; Brusca et al., 2016
Lophophorata	Mature ganglia in several species are epithelial In neurogenesis invagination of the neuroectoderm occurs	Has great variations between species Possess one or two well organized giant nerve fibers Composed by a dorsal and an inner ganglion, an inner and a tentacular nerve ring, and six nerves per tentacle	Most nerves associated with the body wall (intraepidermal or immediately subepidermal) Supplies the tentacles with nerves and some motor nerves originate the longitudinal muscles	Temereva and Malakhov, 2009; Hartenstein and Stollewerk, 2015; Brusca et al., 2016
Annelida	Ectoderm domains present cylindrical epithelial cells with increased proliferative activity that originate nervous cells	Distributed in two connectives and in four transverse commissures Formed by the ventral nerve cord, by a ganglion, and by nerves located within each segment Two glial cells are present in some species with processes of 0.3–0.9 μm thick	Nerves innervate the tissue form the body wall to the digestive tract Inside each medium-ventral segment, nerves are responsible for the impulse transmission from the motor nerves to the sensory nerves	Purschke, 1990; Orrhage, 1993; Orrhage and Müller, 2005; Denes et al., 2007; Rimskaya-Korsakova N.N. et al., 2018
Mollusca	Result of an anterior circumenteric arrangement of ganglia and paired ventral nerve cords Neurogenesis is derived from ectodermal placode where proliferating neural progenitors are packed	Constituted of five (cerebral, buccal, pleural, pedal, and visceral) or six (the five above plus the parietal pair) sets of ganglia (Budelmann, 1995; Bulloch and Ridgway, 1995) Some have glia cells either small (4–5 μm of diameter, 0.2–0.5 μm thick), or large (100–600 μm long, 2 μm thick, 20 μm wide)	Forms highly specialized sensory structures Responsible of the function of sensory tentacles, statocysts, photoreceptors, and osphradia (patches of sensory epithelium near the gill or the mantle wall)	Willmer, 1978; Goldstein et al., 1982; Jacob, 1984; Paemen et al., 1992; Budelmann, 1995; Bulloch and Ridgway, 1995; Coles, 2009; Brusca et al., 2016
Hemichordata	An invagination of ectoderm form the neurochord Initial neurogenesis is not restricted along the dorsoventral axis, and allow the formation of dorso-ventral neurons	Primitive NS with nerve cells distributed throughout the epidermal layer Nerve fibers are disposed in two layers: one thin basal layer composed of fibers with an irregular arrangement, and one thicker superficial layer whose fiber organization are forming the conducting system	Regulate sensory cells (located over most of the body), the preoral ciliary organ, and the touch receptors	Knight-Jones, 1952; Lowe et al., 2006; Cunningham and Casey, 2014; Brusca et al., 2016
Echinodermata	Noticeable nerves are derived from the ectoneural system Neurogenesis is regulated by its basiepithelial nerve plexus, where packed neurons form cords or ganglia	Present a relatively simple architecture with a radiate pattern Is made up of three networks (ectoneural or oral, hyponeural, and entoneural or aboral) combined into ganglionated nerve cords Organized in a circular or pentagonal circumoral nerve ring Glia cells are 45–70% of the total NS cell mass	Plexus of the ectoneural system innervate the simple epithelial sensory receptors Epidermal sensory neurons are sensitive to water currents, light, touch, and dissolved chemicals	Hyman, 1955; Bellock, 1965; Pentreath and Cobb, 1972; Cobb, 1987; García-Arrarás et al., 2001; Mashanov et al., 2010; Brusca et al., 2016

*The table shows the known main characteristics for each Phylum in terms of development, neurogenesis, anatomy, and physiology.*

### Protostomia

#### Nematoda

The NS of Nematoda is simple and accessible, and is constituted by a group of a few hundreds of neurons whose number ranges between 194 in *Pellioditis marina* and 302 in *Caenorhabditis elegans* (Houthoofd et al., 2003; Blaxter, 2011). In nematodes the cell lineage and configuration of NS is not fixed, and is constantly adjusted according to the organism functional necessities. During development, nematode neuron’s final position is close to the place where they originated as has been observed in the marine organisms *Enoplus brevis* and *P. marina* (Voronov and Panchin, 1998; Houthoofd et al., 2003). The study of the NS in this group of organisms has shown that the development of this system, together with the epidermis and the pharynx, between terrestrial and marine annelids is different and present low similarity. These were shown when comparing tissue configuration of *C. elegans* and *P. marina* embryos, a low similarity degree between their NS was observed (Houthoofd et al., 2003). Due the similarity between NS organization in organisms with big brains and the neural circuits in nematodes, these organisms have been used as model (Schafer, 2016) of nervous processes. Such is the case of *Nematostella vectensis* for neurogenesis (Layden et al., 2016), and *Deontostoma californicum* for the study of the NS organization (Siddiqui and Viglierchio, 1971, 1977).

#### Arthropoda

Nervous system of arthropods is constituted by the dorsal brain, and by a ventral, longitudinal, and ganglionated nerve cord. The nerve cord is primitively paired and is the origin of all lateral nerves that extends along each segment of the arthropod body (Laverack, 1987). The NS of this group of marine invertebrates is similar to the annelids. Due to their similarities, it was thought that arthropods evolved from annelids, but molecular evidence is suggesting that they are evolved independently (Harzsch, 2006; Wolfe et al., 2019). The NS of arthropods involves the body surface where are the distinct sensillia (sense) organs like leg tips, joints, mouthparts, and antennae (Laverack, 1987; Elofsson and Hessler, 2005). The arthropod’s sensory system is highly organized, and presents a specific architecture in each genus. These organisms are growing continuously and have numerous molts during their adult life. Each molt must have new sensillia organs which combine with the old ones, resulting in an increasing and complex nervous net as long as they live. Study of cellular anatomy and architecture of arthropods NS, besides their physiology, requires specific investigations to get a better comprehension of how it is working (Benzid et al., 2005; Brenneis and Richter, 2010). In neurosciences, particular characteristics of arthropod’s NS has been studied mainly to get a better understanding of neurogenesis, besides to know a distinct type of neuron-muscle communication.

Arthropod’s small size muscles generate a peculiar neuromuscular organization. As result, muscular contractions are regulated by the specific neuron type on charge of each one of the muscular fibers (Harzsch, 2006). Arthropod muscular innervation is multiterminal and polyneuronal with mostly monopolar motor neurons. The interneurons of arthropods do not produce action potentials, do not drive the postsynaptic neurons by transmitter release, and some are non-spiking; this is probably related with the short distance between neurons in a ganglion. In arthropods like in other invertebrates, one motor neuron innervates multiple muscle fibers, and the primary excitatory neurotransmitter involved is glutamate, while vertebrates use acetylcholine. As well, muscle contraction is mediated by γ-amino-butyric acid (GABA), which binds to muscle fibers that receive inhibitory synaptic information (Smarandache-Wellmann, 2016). In neuron-muscle communication both vertebrates and arthropods express transcription factors as Foxf1, myocyte-specific enhancer factor 2 (Mef2), and Nkx3.2 (Arendt et al., 2016). This makes arthropods interesting models to understand the functioning of neurons involved in processes as simple as bending a limb, or more complex such as sensing the environment or emitting snaps. For example, in *Limulus polyphemus* leg contraction is the result of six axons of excitatory neurons that are innervating the muscle (Parnas et al., 1968; Wyse and Dwyer, 1973). On the other hand, a great axon number is necessary to perform the distinctive snaps of each shrimp’s genus. In *Alpheus heterochelis* and *Aplysia californiensis* the snapping mechanisms are regulated by both motor and sensory neurons, but there each one involves distinct axon numbers (from 3,000 to 13,000) (Knowlton and Moulton, 1963; Ritzmann, 1974; Read et al., 1991). In terms of neurogenesis, animals like the crab *Carcinus maenas* (Hansen and Schmidt, 2001), and the sea spider *Pseudopallene* sp. (Brenneis et al., 2013) are being used mainly as models to understand the process during the embryonic development. Nevertheless, a novel approach is being given to the capacity of adult arthropods to generate new-born neurons. Evidence suggests that in adult organisms of distinct phylum, including arthropods, neurogenesis is regulated by a common and ancestral mechanism (Sandeman et al., 2011). It is not clear yet what is the functional and applied contribution of this knowledge to the human health, but the generation of new neurons in adults organism, is always an important issue that deserve a detailed research.

#### Lophophorata

Lophophores are organisms with unique structural patterns, and as adults they live in colonies. Their NS organization has great variations between species. Lophophorata possess one or two well organized giant nerve fibers, and do not have a clear organization in neither the nerve trunks nor the nerve ganglia (Temereva and Malakhov, 2009). Lophopore NS is composed by a dorsal and an inner ganglion, an inner and a tentacular nerve ring, and six nerves per tentacle; these structures are controlled by three distinct nerve centers named dorsal-ganglion, outer lophophoral nerve ring, and inner lophophoral nerve ring (Temereva, 2017). Variations in NS of lophophores are related with the organization of their distinctive structures (tentacles innervation and their connection with the main nerve cores, and cerebral ganglion) (Temereva and Kosevich, 2016). The study of lophophore adult innervation is useful to clarify its monophyly origin, which still is confusing. Until now, exist few research about the physiology of the NS in this class; almost all the investigations are focused in understanding the origin of its NS, and its relationship with other phylum. *Phoronopsis harmeri* (Temereva and Malakhov, 2009), *Symbion pandora* (Wanninger, 2005), *Phoronis australis* (Pardos et al., 1991), and *P. harmeri* (Temereva and Tsitrin, 2014) are some of the lophophore models used to elucidate the anatomy, architecture, and functioning of the NS of these peculiar organisms.

#### Annelida

In annelids, the NS present ancient and distinctive characteristics distributed in two connectives and in four transverse commissures. Polychaeta is the first and largest class of Annelida (Dales, 1967). Their nerve structures have been found and reported in almost all studied families of Polychaeta for decades (Wells et al., 1972; Günther, 1975; Orrhage and Eibye-Jacobsen, 1998; Rimskaya-Korsakova N.N. et al., 2018). The NS of annelids is formed by the ventral nerve cord or medium-ventral segment (composed of two strands that extends to the length of the organism), by a ganglion, and by nerves located within each segment (Orrhage and Müller, 2005). In each segment, nerves innervate the tissue form the body wall to the digestive tract (Orrhage, 1993; Rimskaya-Korsakova N. et al., 2018). Inside each medium-ventral segment, nerves are responsible for the impulse transmission from the motor nerves or ganglion, to the sensory nerves or receptor. Surface epithelium tissue of these organisms has the cell bodies of sensory nerves, meanwhile inside either the ganglion or separate parapodial ganglia, are located the motor nerves. In the majority of the annelids, their nerve cord has giant fibers called neurochords. Rapid impulses transmission from one end of the worm to the other are carried out by these neurochords, which in some genus like *Myxicola*, measure 1.5 mm in diameter and are not covered with myelin (Wells et al., 1972; Eng et al., 1980; Orrhage and Müller, 2005). Nevertheless, individual nerve cells of their discrete ganglia are not always easily observable under the microscope (Blackshaw, 1986).

Characteristics of annelids make them important models for the study of different features of the NS. Among these excels the study of nerve regeneration processes in distinct Polychaeta genus. Regeneration has been studied mainly in *Nereis virens* (Holmes, 1931), *Nereis diversicolor* (Clark and Bonney, 1960), *Owenia fusiformis* (Coulon et al., 1989), *Eurythoe complanata* (Zapata-Vívenes, 2005), *Dorvillea bermudensis* (Paulus and Müller, 2006), *Cirratulus* cf. *cirratus* (Weidhase et al., 2014), *Typosyllis antoni* (Weidhase et al., 2017), *Alitta virens* (Kozin et al., 2017), and in *P. dumerilii* (Kostyuchenko et al., 2019). Collectively, these studies demonstrated that each specie present differences in its ability to regenerate. Moffett (1996) and Müller et al. (2003) considered that these results are probably due to (1) the intrinsic growth program of each annelid species and (2) the relationship between the wound done experimentally in the worms, the distance to the ventral cord, and the intensity of the signals that must be transmitted by the ventral cord.

#### Mollusca

Molluscs are a large and discrepant collection of organisms. Marine species are classified into five groups that present great metabolic and phenotypic diversity. This variability includes the NS, which in squid and octopus is as complex as that of lower invertebrates; meanwhile in monoplacophors and chitons it can be as simple as that of annelids (Budelmann, 1995). Despite the discrepancies between molluscs, the NS of all Phyla shares few similarities. Their NS is constituted of pairs of ganglia, which are divided in five (cerebral, buccal, pleural, pedal, and visceral) or six (the five above plus the parietal pair) sets (Budelmann, 1995; Bulloch and Ridgway, 1995). Molluscs also have highly specialized sensory structures, like the aesthetes in chitons or the sensory tentacles in cephalopods (Sigwart and Sumner-Rooney, 2016). The molluscs study has not only contributed to a better understanding of neuronal functioning and its evolutionary process, but has also served for the phylogenetic classification of some species, which previously were not known to be molluscs (Sigwart and Sumner-Rooney, 2016).

In neurosciences, *A. californica*, a marine mollusk, has been an important model organism to the study of neuron functions (Frazier et al., 1967; Kriegstein, 1977) due the facility to identify its nervous cells by their position, their size, and their electrophysiological properties. Neurons of *A. californica* have allowed the characterization of neuron nuclear DNA and chromatin arraignment from individual neurons (Coggeshall et al., 1971; Lasek and Dower, 1971; Treistman and Schwartz, 1976), besides the study of the RNA metabolism in neurons (Berry and Cohen, 1972), its transcriptome (Moroz et al., 2006), and the synthesis of specific proteins in single phenotypically different neurons (Loh and Peterson, 1974). The characterization of neurons containing choline (Giller and Schwartz, 1971), serotonin (Ono and McCaman, 1984; Marois and Carew, 1997), and its function in synaptic transmissions (Pellmar and Carpenter, 1980; Mauelshagen et al., 1998), as well as the role of other peptides in motor neurons (Church and Lloyd, 1991, 1994; Whim et al., 1993), has been possible too in the sea slug. The neuron research in *A. californica* has led to the development of the L7 neural cell line from its abdominal ganglion, that have served for the study of the metabolite content and the osmolyte of single neural cells (Grant et al., 2000). Characteristics of this kind of mollusc’s brains have allowed even molecular studies related to the consolidation of memory and learning in *A. californica* (Kandel and Schwartz, 1982; Kandel, 2006), and using another sea slug named *Hermissenda crassicornis* (Tamvacakis et al., 2015).

Cephalopods are an unusual kind of Mollusca of great interest too in the field of neurosciences due the higher functions of their NS, which are similar to vertebrate and present a blood–brain barrier of glia cells (Bundgaard and Abbott, 1992; Budelmann, 1995; Maselli et al., 2018). In the cephalopod brain of organisms like sepia and octopus, recognized neurotransmitter molecules has been found, in addition of high efferent innervation of receptor cells and afferent first-order peripheral neurons (Bullock, 1984; Bullock and Başar, 1988; Bullock and Budelmann, 1991; Budelmann, 1995).

### Deuterostomia

#### Hemichordata

Hemichordates are organisms with a bilateral symmetry during all their lifespan. They live either solitary or in filter-feeding colonies, and are key animals in the evolution field to investigate the origins of the clade Deuterostome. Their closer relationship with echinoderms and chordates generated that Hemichordata receive attention since the last century (Dakin, 1916; Knight-Jones, 1952). Evidence suggests that chordates and hemichordates share the ontogenetic module that originated the NS at not the same parts of the body (Nomaksteinsky et al., 2009). Hemicordata has a primitive NS with nerve cells distributed throughout the epidermal layer. Nerve fibers are disposed in two layers: the first one is a thin basal layer composed of fibers with an irregular arrangement, and the second one is a thicker superficial layer whose fiber organization are forming the conducting system (the main nerve cord and the small bundles) (Knight-Jones, 1952). Hemichordates are fragile organisms that present a remarkable and primarily epimorphic regeneration capacity which includes their nerve net (Rychel and Swalla, 2009; Luttrell et al., 2016). Evidence of its regeneration ability has been proved in solitary hemichordates, since there is little information for colonial hemichordate regeneration. Due their bilateral symmetry as adults, hemichordates show analogous regeneration patterns with the chordates. This has been proved at molecular level, where gene expression analysis revealed that Hemicordata regeneration requires an independent stem-cell endogenous transdifferentiation, and particular genetic mechanisms (Lowe et al., 2003; Arimoto and Tagawa, 2015, 2018; Miyamoto and Wada, 2015). In Deuterostome, Hemichordate are the only known to regenerate the complete NS of the anterior head-like structure, as *Ptychodera flava* that has the ability to regenerate their entire NS in 1–4 days (Humphreys et al., 2010; Luttrell et al., 2016). Besides *P. flava*, *Glossobalanus berkeleyi* is being used too to study the molecular bases of the hemichordate regeneration process (Rychel and Swalla, 2009). As long as *Ptychodera bahamensis* and *Saccoglossus kowalevskii* are neurulation models in which it has been possible to conclude that in the collar cord some neuronal integration occurs (Kaul and Stach, 2010). Despite what we know so far, there are only few hemichordates that are used as neuronal models. It is necessary to carry out more studies in distinct hemichordates to get a better understanding of the NS regeneration mechanisms, and elucidate how to apply this in the neurosciences field.

#### Echinodermata

In metazoans, Echinodermata is considered one key phylum in evolution and ecology studies, but it its NS is one of the least studied due to the technical difficulties. Despite this, NS of echinoderms has been studied during the past 75 years (Hyman, 1955; Bellock, 1965; Pentreath and Cobb, 1972; Cobb, 1987; García-Arrarás et al., 2001; Mashanov et al., 2013; Díaz-Balzac and García-Arrarás, 2018), and important information has been added to the understanding of these organisms. The close phylogenetic relationship between echinoderms and chordates, and the evidence that they shared a common ancestor (Winchell et al., 2002; Blair and Hedges, 2005), make them a unique study model in neurosciences. Echinoderms’ NS present a relatively simple architecture with a radiate pattern be made up of three networks (ectoneural or oral, hyponeural, and entoneural or aboral) combined into ganglionated nerve cords (Hyman, 1955; Pentreath and Cobb, 1972; Cobb, 1987; García-Arrarás et al., 2001). In five of the six phylum of echinoderms (Crinoidea, Asteroidea, Ophiuroidea, Echinoidea, and Holothurioidea), the ectoneural system constitutes the main component of their NS; in adults of class Crinoidea, aboral system controls them (Hyman, 1955; García-Arrarás et al., 2001). Motor, sensory, and interneurons, besides radial glia cells, have been identified in the central NS of echinoderms. Transmission in both motor and sensory neurons is mediated by acetylcholine; whereas that in the interneurons, the neurotransmitters are noradrenaline and/or dopamine. Axons of the Echinodermata neurons are small and unmyelinated, and are disposed in packages with a parallel arrangement. In the plexus and on the surface of the primary podia, epithelial (sensory) cells are responsible of axon supply. The distinct types of axons sometimes are not easily distinguishably, such is the case of the peripheral axons which can be confused with the processes of muscle and interstitial cells (Pentreath and Cobb, 1972; Byrne et al., 2018; Hinman and Burke, 2018).

In neurosciences, echinoderms are an extraordinary model for the study of neuronal regeneration processes. They have the remarkable capacity for regrown both external and internal parts, including the NS. Molecular studies revealed interesting aspects related with the neuroregeneration in Echinodermata like the presence of pluripotency factors orthologs, known in mammalian cells as Yamanaka factors (Mashanov et al., 2015a); the expression of *Hox* gene homologs (Thorndyke et al., 2001); and the involvement of growth factors (TGF-β, NGF, and FGF-2), neuropeptides (substance P), and neurotransmitters (monoamines) in the regeneration process (Thorndyke and Candia Carnevali, 2001). It is remarkable the possibility to carry out this kind of studies in all class of the Phylum Echinodermata. There are evidence of NS regeneration in Asteroidea species as *Marthasterias glacialis* (Franco et al., 2012), *Asterias rubens* (Moss et al., 1998), and *Ophiocoma echinata* (Pomory and Lawrence, 1999); in Ophiuroidea like *Ophioderma longicaudum* and *Amphiura filiformis* (Dupont and Thorndyke, 2006; Biressi et al., 2010); in *Eupentacta fraudatrix* (Mashanov et al., 2008), and *Cladolabes schmeltzii* (Kamenev and Dolmatov, 2015) class Holothuroidea; in the Echinoidea *Psammechinus miliaris* (Chia, 1970); and in the Crinoidea *Antedon mediterranea* (Candia Carnevali et al., 1997). In all cases, evidence indicates that during the lifespan of these organisms an unusual NS regeneration program can be activated by distinct neurotrophic factors that include morphogens, mitogens, and growth and/or regulatory molecules.

## Role of Glia Cells in Marine Invertebrates

Glia cells are defined as non-neuronal cells of the NS, with particular biochemical and structural characteristics, that allow them to get involved in all those functions that are carried out in the NS (Zhang, 2001). The evidence suggests that in the processes of evolution, development and regeneration, glia cells have an important role. Invertebrate glia cells share both morphological and physiological characteristics with vertebrates (Tables 2, 3). As in chordates, glial cells of marine invertebrates participate in osmoregulation, neuroprotection, neurogenesis, and neuroregeneration processes. Nevertheless, in some particular cases the role of glia cells is not clear and new research is required. In a similar manner as in mammals, in invertebrates glutamate is considered a necessary substance to cellular signaling between glia cells and the axon (Lieberman and Sanzenbacher, 1992). Glutamate effect has been demonstrated in the hyperpolarization of Schwann cells of squids, mainly in *Alloteuthis* sp. and *Loligo* sp. (Lieberman et al., 1989; Inoue et al., 2002). In *Aplysia*’s sensorimotor synapses glutamate act as a neurotransmitter, and glutamate transporters has been located within similar to mammalian disposition (Levenson et al., 2000).

**TABLE 2:** Characteristics of glial cells in marine invertebrates and vertebrate organisms.

Characteristic	Marine invertebrates	Vertebrate organisms	References
Shape	Ovoid, symmetrical and asymmetrical forms	Ovoid, star-shaped, symmetrical and asymmetrical forms	Siddiqui and Viglierchio, 1977; Auld and Robitaille, 2003; Orrhage and Müller, 2005
Soma range	4–20 μm, 0.2–2 μm thick	1.1–50 μm	Willmer, 1978; Goldstein et al., 1982; Paemen et al., 1992; Rajkowska et al., 1998; Coles, 2009
Nuclear shape	Rounded or ovoid	Rounded, ovoid, elongated, comma shaped, and polylobular	Mashanov et al., 2013; García-Cabezas et al., 2016
Cytoplasm	Usually not visible	Usually not visible	García-Cabezas et al., 2016; Mashanov and Zueva, 2018
Process length	Up to 600 μm	Up to 300 μm	Willmer, 1978; Goldstein et al., 1982; Paemen et al., 1992; Coles, 2009; Kettenmann and Verkhratsky, 2011

*The table summarizes the known glia characteristics of marine invertebrates in which these cells have been found.*

**TABLE 3:** Glia functions in marine invertebrates.

Phylum	Reported species	Glia functions	References
Arthropoda	*Limulus polyphemus*	Osmoregulation	Harrison and Lane, 1981
	*Homarus vulgaris*	Nutrition of synaptic region	Hámori and Horridge, 1966
	*Glyptonotus antarcticus*, *Panulirus argus*, *Panulirus interruptus, Carcinus*	Formation of axon sheath	Horridge and Chapman, 1964; Laverack and Ardill, 1965; Spencer and Linberg, 1986; Chaigneau et al., 1991
	*Pseudopallene* sp.	Embryonic neurogenesis	Brenneis et al., 2013
Annelida	*Armandia brevis* and *Protodrilus* sp.	Encapsulation of light-receptive sensory organs	Hermans, 1969; Purschke, 1990
	*Myxicola infundibulum*	Formation of axon sheath	Wells et al., 1972
	*Nereis diversicolor*	Chemotaxis and possible protection against cellular compression	Baskin, 1971; Paemen et al., 1992
Mollusca	*Mactra stultorum*	Possible uptake and inactivation of neurotransmitters	Elekes, 1978
	*Mytilus edulis*	Osmoregulation	Paemen et al., 1992
	*Aplysia* sp.	Intracellular transport of macromolecules and calcium homeostasis	Goldstein et al., 1982; Maggio et al., 1991
Echinodermata	*Holothuria glaberrima*	Precursors of new-born glial cells and neurons	Mashanov et al., 2008, 2013; San Miguel-Ruiz et al., 2009
	*Sphaerechinus granularis*	Possible participation with sensory system of epidermal cells	Flammang and Jangoux, 1993

In marine invertebrates, glia cells are reported as “generally absent” but several investigations have proved that these cells are present in numerous marine phylum. The number, kind, and distribution of glial cells in the NS of invertebrates are variable. For example, some marine nematodes and annelids species have two glial cells with processes of 0.3–0.9 μm thick (Teuchert, 1977; Purschke, 1990). In certain molluscs glia cells are small (4–5 μm of diameter), and their filaments are 0.2–0.5 μm thick; while in other molluscs like the giant squid, glia cells are 100–600 μm long and approximately 2 μm thick, 20 μm wide (Willmer, 1978; Goldstein et al., 1982; Paemen et al., 1992; Coles, 2009). In echinoderms, glia cells can compose 45–70% of the total NS cell mass (Mashanov et al., 2010). Although the presence of glial cells has already been reported in almost all Phyla of marine invertebrates (exceptions are Lophophorata and Hemichordata), the function that these cells have in some of the phylum (like Nematoda) is still unknown, or its literature is not obtainable. It is possible that in some cases glia cells are very little and only few commercial cell markers are specific for marine invertebrates, like has been reported in *Aplysia* (Lockhart et al., 1996). In the following paragraphs and in Table 3, the known functions of glial cells for marine invertebrates are mentioned.

Arthropods as *L. polyphemus* uses its glial cells for osmoregulation; if it is true that glial cells are not forming a blood-barrier like in insects, exist junctional complexes between them to restrict the entry of ions and molecules (Harrison and Lane, 1981). Glia cells also participate in the nutrition of the synaptic region in the lobster *Homarus vulgaris* (Hámori and Horridge, 1966). In *Glyptonotus antarcticus* these cells are wrapping its sensory neurons (Chaigneau et al., 1991), which is similar in *Panulirus argus* and *Panulirus interruptus* sensory cell bodies, and in the leg nerves of the crab *Carcinus*, where glia cells form a sheath (Horridge and Chapman, 1964; Laverack and Ardill, 1965; Spencer and Linberg, 1986). As well, in early embryonic stages of *Pseudopallene* sp. immature glia cells are participating in neurogenesis (Brenneis et al., 2013).

In annelids as far as glial cells are concerned, they have different functions in each one of the annelid species where they have been found. In *Armandia brevis* processes of sensory nerves are projected into the ocellar cavity, where they almost filled it; the cavity is lined by squamous glial cells which enclosed the photoreceptors in the prostomium (Hermans, 1969). In *Protodrilus* sp. processes of glial cells are encapsulating its light-receptive sensory organ (but its exact function is still unknown) (Purschke, 1990). In *Myxicola infundibulum* glial cells are forming a sheath that surrounds the giant axon (Wells et al., 1972). Meanwhile in *N. diversicolor* is thought that glia cells can induce chemotaxis (Paemen et al., 1992) or may be protecting the neurons from cellular compression (Baskin, 1971).

In Mollusca, different glia cells have been discovered in the same organism. In *Mactra stultorum* it is suggested that glial elements are participating in transmitter uptake and inactivation (Elekes, 1978). *Mytilus edulis* has at least two types of small glia cells (Paemen et al., 1992), whose are participating in osmoregulation processes to keep the organism’s osmotic and ionic equilibrium (Willmer, 1978). In *Aplysia* glia cells are contributing to the intracellular transport of macromolecules, and to the intracellular glial calcium homeostasis (Goldstein et al., 1982; Maggio et al., 1991).

Despite the recognized ability of echinoderms to regenerate their NS, even during their life as adults, there are very few species in which the function of glia cells has been reported. Of relevance are the studies reported Mashanov and co-workers in the NS of the sea cucumber (Mashanov et al., 2007, 2009, 2010). They described that in echinoderms, radial glia cells are the major and unique type of glial cells. Radial glial cells of *Holothuria glaberrima* adults, act as precursors of new-born glial cells and neurons (San Miguel-Ruiz et al., 2009; Mashanov et al., 2013), and constitute the supporting scaffold during neuronal migration (Mashanov and Zueva, 2018). Molecular aspects of this process regulated by glia cells showed that *Myc* and *Bmi-1*, besides mammal’s gene homologs like *Sox2*, *Oct4*, and *Klf4* take part in sea cucumber neuroregeneration (Mashanov et al., 2015b, 2014). Besides their role in neuroregeneration, echinoderm’s glial cells processes are associated with epidermal sensory cells of *Sphaerechinus granularis*, a sea urchin (Flammang and Jangoux, 1993).

## Conclusion

Marine invertebrate organisms live in a great variety of ecosystems, where are exposed to drastic ambient changes. For example, animals living in shores requires very particular homeostatic systems to regulate their metabolism and avoid physiologic damages as consequence of temperature, atmospheric pressure, nutrients availability, and sea water salinity variations that occur during tidal changes. As many organisms, marine invertebrates are susceptible to predation, so they need run away mechanisms to ensure their survival. Each one of these survival requirements should be controlled by the NS, which is relatively less complex in invertebrates and therefore theoretically easier to study. In almost all marine invertebrate phylum, the presence of neurons and glia has been demonstrated. In addition, the anatomy and architecture of the NS of many marine invertebrates has been described for more than a century, so there is a significant amount of information about it. For some organisms the function of both cell types has also been described. In other cases and as mentioned above, further studies are required. These reasons all together make marine invertebrates excellent model organisms for the neurosciences field.

Diverse marine models are being already used to understand important process in the NS as neurogenesis, neuroregeneration, neuroprotection, and neuro-osmoregulation. Among them, are distinguished the sea cucumber *H. glaberrima*, used to understand NS regeneration after an injury or deletion; the squid giant *Architeuthis* and the sea slug *Aplysia*, which have been used to study *in situ* the interactions between neurones and glia cells in the context of complex brain functions; the mussel *Mytilus* for the study of NS homeostatic control; *Nereis* to the study of nerve regeneration processes; *Alpheus* to understand the snapping mechanisms regulated by both motor and sensory neurons; *Limulus* to delineate neuron control on leg contraction, besides NS osmoregulation; *Panulirus* to be familiar with glial neuroprotection; *Deontostoma* and *Nematostella* for or the study of the NS organization; *Pseudopallene* sp. for embryonic NS development.

Although higher vertebrates have unique cerebral abilities, the physicochemical principles of all brains follow basic and in some cases common strategies. These include organization, neuron-glia communication, gene expression, release and transport of neurotransmitters and other molecules, osmoregulation, formation of glial sheaths, among others. To the extent that the common characteristics between the NS of the chordates and the non-chordates are known and better understood, it will be possible to generate knowledge about the correlation that exists in processes as particular as neuronal regeneration. Once this is achieved, it will be possible to apply such knowledge for the treatment of neurodegenerative diseases in humans and with this, improve their quality of life.

## Author Contributions

AO contributed to the writing, editing, and discussion of this contribution. TO-B structured and wrote the manuscript.

## Conflict of Interest

The authors declare that the research was conducted in the absence of any commercial or financial relationships that could be construed as a potential conflict of interest.
